# Successful selective reduction of a heterotopic cesarean scar pregnancy in the second trimester: a case report and review of the literature

**DOI:** 10.1186/s12884-016-1171-x

**Published:** 2016-11-29

**Authors:** Haiyan Yu, Hong Luo, Fumin Zhao, Xinghui Liu, Xiaodong Wang

**Affiliations:** 1 Department of Obstetrics and Gynecology, West China Second University Hospital, Sichuan University, Chengdu, China; 2 Department of Ultrasonic Medicine, West China Second University Hospital, Sichuan University, Chengdu, China; 3 Department of Radiology, West China Second University Hospital, Sichuan University, Chengdu, China; 4 Key Laboratory of Birth Defects and Related Diseases of Women and Children (Sichuan University), Ministry of Education, No.20, 3rd section, South Renmin Road, Chengdu, Sichuan 610041 China

**Keywords:** Heterotopic cesarean scar pregnancy, Potassium chloride, Selective fetal reduction, Expectant management

## Abstract

**Background:**

Heterotopic cesarean scar pregnancy is a cesarean scar pregnancy combined with an intrauterine pregnancy that predisposes a woman to life-threatening complications such as uterine rupture and massive bleeding. Preservation of the intrauterine pregnancy in heterotopic cesarean scar pregnancy is a great challenge.

**Case presentation:**

We report a case of a 33-year-old woman with heterotopic cesarean scar pregnancy after IVF-embryo transfer (ET). Expectant management was carried out with early diagnosis of heterotopic cesarean scar pregnancy (HCSP), and selective fetal reduction of cesarean scar pregnancy (CSP) was performed by ultrasound-guided intrathoracic injection of potassium chloride (KCl) at 16 + 4 weeks of gestation due to aggravation of CSP. Preservation of the intrauterine pregnancy was successful and a healthy baby was delivered by cesarean section at 37 + 6 weeks of gestation.

**Conclusions:**

Heterotopic cesarean scar pregnancy is an extremely rare form of heterotopic pregnancy. Patients should be appropriately counseled regarding the different treatment options available. An ultrasound-guided injection of potassium chloride may constitute a safe, minimally invasive and reliable way to terminate the heterotopic gestation and preserve the intrauterine pregnancy. Intensive management should be performed during the ongoing pregnancy and cesarean section.

**Electronic supplementary material:**

The online version of this article (doi:10.1186/s12884-016-1171-x) contains supplementary material, which is available to authorized users.

## Background

Cesarean scar pregnancy (CSP) is one of the rarest forms of ectopic pregnancy, located in the scar of a previous cesarean section. The incidence of CSP has been estimated to be between 1:2216 and 1:1688 [1–4]. CSP results in life-threatening complications such as uterine rupture and catastrophic hemorrhage, which may, in turn, be associated with maternal and fetal morbidity and mortality [5]. Therefore, immediate intervention (sometimes including hysterectomy), must be performed [6].

Heterotopic pregnancy (HP) is defined as the simultaneous presence of intrauterine pregnancy and ectopic pregnancy, which is very rare but a potentially life-threatening condition. HP can be spontaneous or the subsequence of assisted reproductive technology (ART), with the frequency of spontaneous heterotopic pregnancy reported as between 1 in 50,000 to 1 in 10,000 [7, 8] and the incidence in ART is 0.2–1% [9].

Heterotopic cesarean scar pregnancy (HCSP) is cesarean scar pregnancy combined with an intrauterine pregnancy that predisposes a woman to life-threatening complications such as uterine rupture and massive bleeding. The incidence of HCSP during spontaneous cycles is extremely low. However, with the increasing incidence of cesarean section and extensive use of assisted reproductive technologies, the prevalence of heterotopic cesarean scar pregnancy (CSP) is expected to rise. Preservation of an intrauterine pregnancy in HCSP is a great challenge, and only isolated case reports of such pregnancies have been published in the literature, with the first case of HCSP published by Salomon et al. in 2003 [10]. Despite the lack of a standard treatment protocol, expectant management and medical and surgical treatment modalities have recently been suggested. In fact, urgent hysterectomy may be required in patients with uncontrolled hemorrhage and uterine rupture.

We used a list of keywords, including “Ectopic pregnancy,” “Cesarean section scar,” and “Heterotopic cesarean scar pregnancy,” to perform an extensive search of the literature; and we found fewer than 25 cases of HCSP having been reported. In the present article, we report 1 case of twin HCSP in a patient after IVF-embryo transfer (ET). We performed transabdominal intrathoracic injection of KCl into a CSP fetus at 16 + 4 weeks of gestation and the intrauterine pregnancy was successfully preserved. In addition, we performed a Medline search and reviewed the English-language literature for similar cases; and these are summarized herein. Written informed consent was obtained from the couple before the procedure and manuscript publication. The treatment procedure followed ethical principles, all data were collected from chart reviews, and approval was obtained from the Institutional Review Board.

## Case presentation

A 33-year-old Tibetan woman, gravida 2, para 1, was admitted because of the abnormal location of one of the two gestational sacs of a twin pregnancy; she had previously undergone transverse lower segment cesarean section 7 years earlier. The patient underwent IVF-ET and 3 embryos were transferred to the uterus; a positive pregnancy test was noted 14 days after embryo transfer. Four weeks after embryo transfer, transabdominal ultrasonography revealed 1 intrauterine gestational sac. However, the patient did not adhere to the physician’s suggestion of a follow-up 1 week later.

**Fig. 1:**
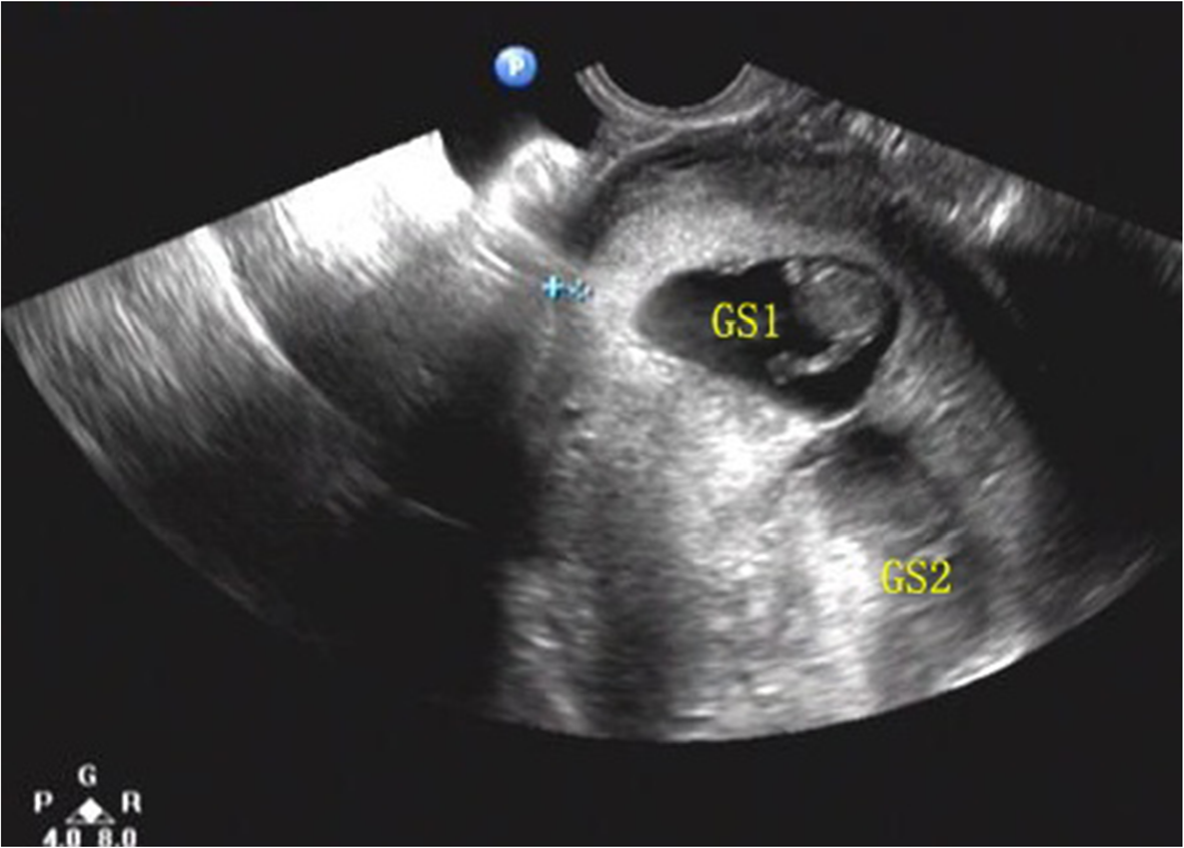
Diagnosis of cesarean heterotopic pregnancy via transvaginal ultrasonography at 11 + 1 weeks’ gestation. Myometrial layer of cesarean scar 4 mm

Sixty days after embryo transfer, transvaginal ultrasonography revealed a dichorionic twin pregnancy with normal cardiac activity and 1 gestational sac situated in the uterine fundus; the other was located lower, immediately over the cesarean section scar, with a thin myometrium of 4 mm in thickness (Fig. 1). The patient manifested no abdominal pain and/or vaginal bleeding. A diagnosis of heterotopic cesarean scar pregnancy was then made, but the couple refused medical intervention.

A follow-up was pursued 7 days later, with both fetuses showing normal fetal cardiac activity and crown–rump length measurements (55 and 48 mm, respectively) that were in accordance with a fetus at 12 + 1 weeks of gestation; 1 fetus was in the upper fundus, whereas the second was still located at the level of the internal os. The ectopic placenta covered the internal cervical os and the cesarean section scar, which was in close proximity to the maternal bladder and which showed the presence of peritrophoblastic vascularity upon Doppler examination.

**Fig. 2:**
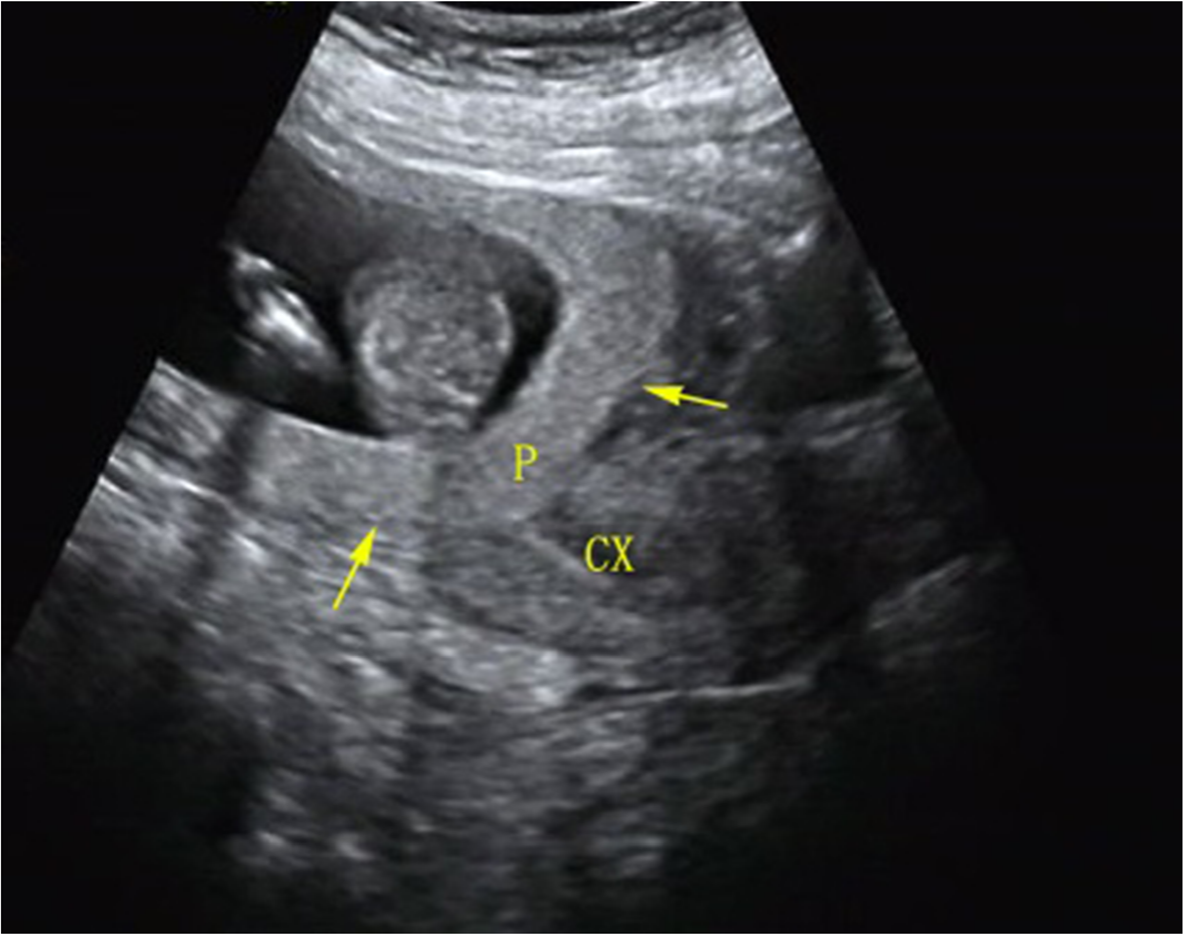
Heterotopic complete placenta previa and placenta accreta at ultrasonography at 16+3 weeks’ gestation

**Fig. 3:**
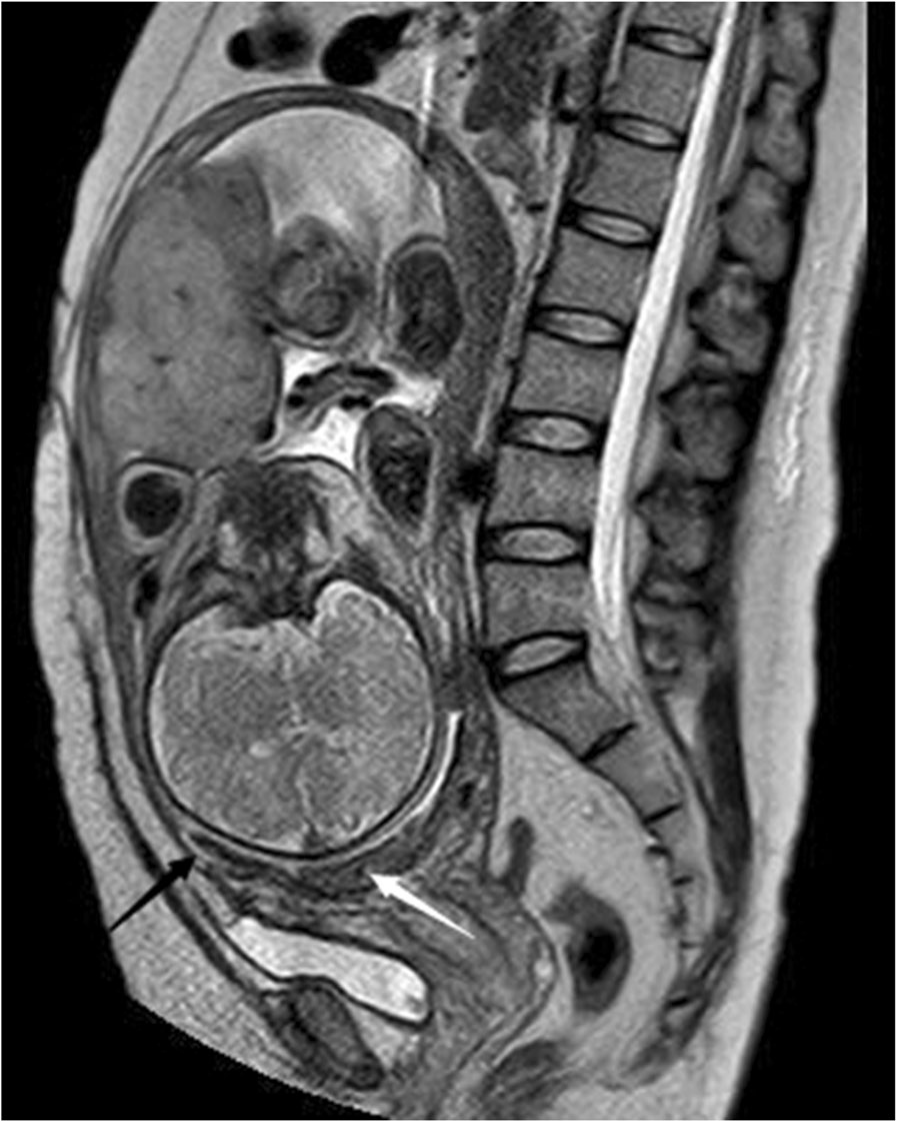
Magnetic resonance imaging findings at 37 weeks’ gestation. Heterotopic complete placenta previa and placenta accrete, dead fetus and placenta covering the internal cervical os (*white arrow*). Thin myometrial layer of cesarean scar (1.3 mm, *black arrow*)

When the patient was transferred to our department, because of the potential of uterine rupture and catastrophic hemorrhage, we extensively counselled the couple regarding the condition, its risks, and the management options for heterotopic pregnancy. We suggested to the couple the use of selective reduction of the heterotopic cesarean scar pregnancy as soon as possible. The couple, however, refused and preferred expectant management; close monitoring was then performed without patient bleeding and lower abdominal pain. At 16 + 3 weeks of gestation, transabdominal ultrasonography revealed a thin myometrial layer of the cesarean section scar (3.8 mm thick) and complete *placenta previa* (Fig. 2). Massive bleeding and/or uterine rupture during an ongoing pregnancy with expectant management was discussed again with the couple, and the patient opted for selective termination of the abnormally located fetus; this was then accomplished at 16 + 4 weeks of gestation by ultrasound-guided intrathoracic injection of 1 mL of 10% KCl using a 20-G needle inserted transabdominally under local anaesthesia with lidocaine. Upon follow-up, ultrasonography was repeated every two weeks. Although fetal biometric parameters of the ongoing pregnancy were normal, the ectopic placenta still showed total *placenta previa* and *placenta accrete*. At 37 weeks’ gestation, a written informed consent was obtained from the couple undergoing MRI to evaluate the placenta. MR image showed heterotopic complete placenta previa and placenta accreta, marked focal thinning of myometrium at the region of cesarean scar. The internal cervical os was covered completely by placenta and dead fetus (Fig. 3).

At 37 + 6 weeks of gestation the baby was delivered by elective cesarean section. Before the operation, we extensively counseled the couple about the possibility of CS hysterectomy due to total *placenta previa* and *placenta accreta*. We noted intraoperatively that profuse vascularization covered the lower uterine segment, that the bladder adhered to the anterior lower segment, and that the mass of HCSP was palpated at the lower uterine segment and bulged toward the vesicoperitoneal reflection. A healthy male baby weighing 2890 gm was delivered through a transverse incision 1 cm above this mass, with Apgar scores of 10 and 10 at 1 and 5 min, respectively. When the dead fetus and placental mass of the HCSP were removed, profuse bleeding due to *placenta accreta* ensued, and bleeding was controlled by partial excision of the anterior lower uterine segment along with myometrial sutures and uterine packing with gauze. Three-and-one-half units of packed red blood cells were then transfused. The postoperative period was uneventful and the patient and the baby were discharged 6 days after the operation. The pathologic results of the excised anterior lower uterine segment revealed *placenta accreta*, showing chorionic villi in direct contact with myometrial smooth muscle fibers.

## Discussion

Cesarean scar pregnancy may have a silent clinical course or present with specific clinical symptoms such as abnormal vaginal bleeding and/or abdominal pain or acute abdominal pain due to uterine rupture. Heterotopic cesarean scar pregnancy (HCSP) is unusual as it is a CSP in combination with an intrauterine pregnancy. Due to its rarity, there is no standard treatment protocol for HCSP.

Early diagnosis of CSP in the first trimester may allow the preservation of viability for the intrauterine fetus and avoid maternal morbidity in HCSP. High-resolution vaginal ultrasonography is the preferred diagnostic method for HCSP, and the mean gestational age at CSP diagnosis is reported to be 7.5±2.5 weeks [11]. Ouyang et al. [4] showed that the gestational age ranged from 5 weeks and 3 days to 7 weeks and 4 days in CSP and from 5 weeks and 6 days to 7 weeks and 4 days in HCSP using transvaginal color Doppler sonography.

Treatment options for cesarean scar pregnancy include open surgery, operative hysteroscopy and curettage, systemic or local methotrexate (MTX), transvaginal embryo aspiration, and potassium chloride injection [10–12]. The great challenge in the management of HCSP is to preserve the concurrent intrauterine pregnancy, which makes the therapeutic management more difficult and may not be the same as management of CSP.

Only a few cases of HCSP have been reported, and there is currently no standard treatment protocol for HCSP. Therefore, in the present study, we conducted a search and review of the literature pertaining to HCSP. To the best of our knowledge, a total of 23 cases of HCSP have been reported in the English literature [2, 4, 10, 12–26], and the results are summarized in Additional file 1: Table S1. The reported treatments included conservative management, fetal reduction by potassium chloride, laparoscopic excision, aspiration of embryonic or ectopic gestational sac, hysteroscopy with directed evacuation, injection of a mixture of MTX and KCl, or laparotomic excision.

### Expectant management

The literature includes seven case reports of HCSP with expectant management [4, 21, 25], only one of which was a spontaneous pregnancy; the others developed after in-vitro fertilization. Five cases exhibited vaginal bleeding and/or abdominal pain and 4 cases delivered live births at 35–37 weeks of gestation. In Bai’s report, during the expectant management severe vaginal bleeding occurred at 8 + 4 weeks of gestation, and this resulted in blood loss anemia (Hb 68 g/L) and a blood transfusion; and subsequently, spontaneous abortion of the CSP occurred at 9 + 1 weeks [21]. Ouyang et al. [4] reported that the success rate with expectant management in cases with a non-viable CSP was 100% (5/5); however, we do not know the maternal morbidity in 3 cases, as 2 cases terminated pregnancy at 14 weeks and 6 months, and 1 case was uneventful at the time of the author’s report (18 weeks of gestation). An emergency cesarean section was performed at 35 weeks in 1 patient due to massive hemorrhage caused by complete placenta previa. Kim et al. reported the only example of HCSP with expectant management and live births of 2 vital babies at 37.3 weeks; however, severe postpartum bleeding due to *placenta accreta* occurred and bleeding was controlled by complete excision of the anterior lower uterine segment along with bilateral uterine artery ligation [25]. Therefore, expectant management might be a choice for HCSP, especially in cases with a non-viable CSP.

### Selective fetal reduction

#### Suction aspiration of cesarean scar pregnancy

Suction aspiration of cesarean scar pregnancy under vaginal ultrasonography is another procedure described in the literature [12, 18, 24]. Gupta et al. performed the procedure at 6 + 3 weeks and suction termination of pregnancy was performed at 13 weeks due to trisomy 13 detected [18]. In Hsieh’s report, treatment by embryo aspiration was done at 6 weeks and the concurrent intrauterine twin pregnancy was preserved successfully with delivery at 32 weeks due to preterm labor [12]. Lui et al. reported the first case of treatment for heterotopic CSP using repeated transvaginal aspiration of the gestational sac (which was complicated by arteriovenous malformation ([AVM]), and cesarean section at 37 weeks of gestation and uterine artery embolization (UAE) due to massive bleeding [24].

### Medical treatment in selective fetal reduction

Salomon et al. reported that the first case of HCSP was successfully treated with potassium chloride under sonographic guidance [10]. Subsequently, eight authors [2, 4, 13, 14, 16, 20, 22, 23] reported selective embryo reduction performed using transvaginal ultrasound-guided KCl injection or potassium chloride and methotrexate (MTX) injection into the ectopic gestational sacs between 6 and 10 weeks of gestation, with no complications arising during the procedures. Most pregnancies were uneventful throughout, except for one patient where some vaginal bleeding and uterine contractions occurred at 28 and 34 weeks, respectively. Among the cases reported, only 1 case was delivered at term, 5 cases at 34–36 weeks, 2 cases at 30–31weeks and 1 case was terminated at 14 weeks. Five patients were complicated with a post-partum hemorrhage, in which one was managed with hypogastric artery ligation and subtotal hysterectomy [22], and 1 with bilateral internal iliac artery ligation [14]. There were eight case reports with potassium chloride injections into the HSCP; interestingly, Litwicka et al. [20] injected a potassium chloride and methotrexate mixture into the ectopic gestational sacs in a triplet, which is the first case of triplet HCSP where 2 gestational sacs implanted in the cesarean scar. The baby’s situation in Litwicka’s report was complicated with Miller syndrome. Although this is a genetic condition inherited as an autosomal recessive trait, potential teratogenic effects of MTX on the intrauterine fetus should be considered in the treatment of local injection into heterotopic pregnancies; and MTX-related teratogenicity to the surviving fetus has been reported [27–29].

When cardiac activity is detected in a CSP, selective embryo reduction *in situ* is normally chosen, and intracardiac injection of potassium chloride is typically used (REF?). In addition, since retained placental tissue subsequent to fetal reduction might lead to obstetric complications, this matter should be discussed with the patient. According to the CSP literature, seven babies have been born prematurely due to premature rupture of the membranes, preterm labor, hemorrhage, abruptio placentae, or uterine rupture.

### Surgical removal of the HCSP

An ectopic pregnancy located within cesarean section scar tissue has a high risk of rupture and bleeding. The major problem after medical approaches may be the difficulty of procuring a strong lower segment in the presence of a concurrent intrauterine pregnancy. Urgent hysterectomy may then be required in patients with uncontrolled hemorrhage and uterine rupture; and in order to improve the perinatal outcome of the intrauterine pregnancy, some authors surgically removed the placental tissue and repaired the myometrium (obviating some side effects of medical treatment). All cases reported thus far carried uneventful prenatal courses and the pregnant women delivered live babies at term. Demirel et al. performed a laparoscopic excision of a scar pregnancy at 6 weeks and 5 days of pregnancy; postoperative follow-up was uneventful and the intrauterine fetus was delivered by cesarean section at 38 weeks of gestation [15]. Wang et al. reported hysteroscopic management of heterotopic cesarean scar pregnancy under ultrasound guidance at 7 weeks of gestation, with the baby delivered at 39 weeks of pregnancy [17]. Armbrust et al. successfully excised a HSP by laparotomy at 7 weeks of gestation, with a subsequent uncomplicated cesarean section and delivery of a healthy baby at 37 weeks of pregnancy [26].

Although surgical procedures are suggested as an alternative treatment for HCSP, complications and risks with surgery should be evaluated, including anesthesia accidents and complications, operative blood loss in anatomic dissection and excision trimming of unhealthy tissues, intrauterine embryo disturbance due to the distending medium used during hysteroscopy, and antepartum spotting or uterine rupture during an ongoing pregnancy.

In the present case we did not choose the surgical approach due to placenta previa, with a major ectopic mass needing to be removed, potentially only increasing procedure-related complications and exposing the intrauterine pregnancy to serious risks. At the time of diagnosis, the lack of symptoms and the couple’s desire provided the option of conservative management. Although the patient was asymptomatic, due to extant placenta previa and placenta accreta and the high risk for bleeding and uterine rupture, we decided at 16 + 4 weeks of gestation to inject KCl selectively into the CSP rather than using a conservative management strategy. This action successfully terminated the CSP and the intrauterine pregnancy was successfully preserved. The serial follow-up ultrasounds still revealed placenta previa and placenta accreta*,* and suggested a local blood circulation; and significant blood loss (1800 ml) occurred during the cesarean section due to the retained trophoblastic tissue. This condition was similar to that described by Wang et al. [14] and by Gyamfi et al. [30]. Although local injection of MTX may reduce the risks of persistent trophoblastic tissue, we did not choose to inject MTX due to the possibility of MTX-related teratogenicity to the intrauterine pregnancy.

## Conclusions

Heterotopic cesarean scar pregnancy is an extremely rare form of heterotopic pregnancy. The desire to preserve the intrauterine pregnancy in cases of HCSP is a great challenge, and no universal management guidelines have been established. The current lack of data with respect to best practices should encourage publication of more individual case reports and the establishment of further multicenter studies in the future. Importantly, patients must be appropriately counseled regarding the different treatment options available.

Based on previously reported cases and our case, injection of potassium chloride may be a safe, minimally invasive and reliable way to terminate the heterotopic gestational sac and preserve the intrauterine pregnancy. As retained gestational tissue may contribute to uterine rupture and massive uterine bleeding during an ongoing pregnancy and cesarean section, intensive management should be performed.

## Additional file

Additional file 1:
**Table S1.** All Reported Cases of Heterotopic Cesarean Scar Pregnancy. (DOCX 34 kb)

## Abbreviations

ART: Assisted reproductive technology
CSP: Cesarean scar pregnancy
HCSP: Heterotopic cesarean scar pregnancy
HP: Heterotopic pregnancy
KCl: Potassium chloride
MTX: Methotraxate
UAE: Uterine artery embolization
