# Supplementary material for: Successful selective reduction of a heterotopic cesarean scar pregnancy in the second trimester: a case report and review of the literature
Source: BMC Pregnancy Childbirth. 2016 Nov 29;16:380. doi: 10.1186/s12884-016-1171-x (PMC5126867; doi:10.1186/s12884-016-1171-x)
Supplement: Additional file 1: — Table S1. All Reported Cases of Heterotopic Cesarean Scar Pregnancy. (DOCX 34 kb) [file 12884_2016_1171_MOESM1_ESM.docx]

Table1. All Reported Cases of Heterotopic Cesarean Scar Pregnancy

| Reference | Age | Previous CS (n) | mode of conception | Presenting  Complain | HCSP | Treatment GA | Treatment style | Outcomes and complication | Weight  (gm) | mass | Blood loss in CS(ml) |
| --- | --- | --- | --- | --- | --- | --- | --- | --- | --- | --- | --- |
| Solomon 2003[10] | 36 | 1 | IVF | No complaint | 1IUP+1CSP | 8 wk | Fetal reduction of CSP with KCl | Live birth at 36 wk d/t PROM by CS.  mass removed during CS | 2800 | morphous bulging mass 3 ×3× 3cm | No data |
| Jurkovic 2003[2] | 36 | 3 | Spontaneous | Not reported | 1 IUP+1 CSP | 7 wk | Fetal reduction  of CSP with KCl | ECS d/t vaginal hemorrhage at 31 wk> 1000 mL. | No data | No data | >1000 |
| Hsieh 2004  [12] | 38 | 2 | IVF | Vaginal bleeding and lower abdominal pain | 2 IUP+1 CSP | 6 wk | Embryo aspiration of CSP | Live births at 32 wk d/t preterm labor | No data | No data | No data |
| Yazicioglu 2004[13] | 23 | 1 | Spontaneous | Scant vaginal bleeding | 1 IUP+1 CSP | 7wk 2 d | Fetal reduction of CSP with KCl | Live birth at 30 wk 3 d d/t PPROM and preterm labor. Abnormally located placenta spontaneous ly detached at 26 wk 6d without bleeding. | 1530 | No data | No data |
| Wang 2007  [14] | 38 | 3 | IVF | No complaint | 1 IUP+1 CSP | 10 wk | Fetal reduction of CSP with KCl, | Live birth at 35 wk by CS d/t preterm labor .  Excision of mass during CS  Blood transfusion and bilateral internal iliac artery ligation performed due to massive bleeding | 1820 | morphous bulging mass  3 ×3× 2 cm | >4,000 |
| Demirel 2009[15] | 34 | 1 | Not available | Dark reddish- brown vaginal discharge | 1 IUP+1 CSP | 6 wk 5 d | Laparoscopic excision of CSP | Profuse bleeding during laparoscopic excision.  Live birth at 38 wk by CS | No data | No | No data |
| Taskın  2009[16] | 24 | 1 | Not available | Vaginal bleeding | 1 IUP+1 CSP | 8 wk 4 d | Fetal reduction of CSP with KCl, | Live birth at 34 wk by CS with preterm labor  Mass removed during CS  Transfusion of 2 packed - red blood cell | 2310 | residual mass | massive bleeding d/t myometrial defect |
| Wang 2010  [17] | 31 | 1 | IVF | Vaginal bleeding | 1 IUP+1 CSP | 7 wk | Hysteroscopic-directed  evacuation of CSP + D&C | Live birth at 39 wk by CS | 3250 | No | No data |
| Gupta2010  [18] | 37 | 4 | IVF | lower abdominal discomfort due to severe OHS | 1 IUP+1 CSP | 6 wk3d | Embryo aspiration of CSP | Termination at 13wk d/t trisomy 13 | / | / | / |
| Duenas- Garcia2011  [19] | 34 | 3 | Spontaneous  with a LING-IUD | No complaint | 1 IUP+1CSP | 5 wk | Induced abortion: 4 doses of intramuscular MTX | medically induced abortion | / | / | / |
| Litwicka 2011[20] | 31 | 1 | IVF | No complaint | 1 IUP+2 CSP | 8 wk | Injection the mixture of MTX+KCl to two CSP | Small vaginal bleeding and uterine contractions at 28 and 34wk  Live birth at 36wk by ECS d/t massive hemorrhage and complete abruptio placenta,  Excision of mass during CS  Transfusion of 2 packed - red blood cell | 1900  (Miller syndrome) | amorphous mass5×3cm,with placental and deciduous tissues in pathologic examination | massive blood loss (no data) |
| Bai 2012  [21] | 37 | 1 | IVF | Vaginal bleeding | 1 IUP+1CSP | 7wk6d | Expectant management | Cardiac activity disappearance of CSP and moderate vaginal bleeding at 8wk 4d,blood transfusion given  Spontaneous abortion of CSP at 9wk1d  Live birth at 36+4 wk by CS d/t preterm labor | 2950 | No | No data |
| Ugurlucan  2012[22] | 34 | 1 | Ovulation  induction | No complaint | 1 IUP+1CSP | 6 wk | TVS guidance KCl injection + gestational sac aspiration | Live birth at 38wk by CS d/t placenta praevia totalis and placenta accrete  Massive bleeding managed by hypogastric artery ligation and subtotal hysterectomy | No data | placenta praevia totalis and placenta accrete | severe PPH |
| Uysal 2013  [23] | 29 | 2 | Spontaneous | No complaint | 1 IUP+1CSP | 8 | TVS guidance fetal reduction of CSP with KCl | Live birth by CS at 35 wk d/t preterm labor with incomplete uterine rupture | 2480 | amorphous mass,  with deciduous material in pathologic examination | No data |
| Liu2014  [24] | 36 | 1 | Ovarian stimulation and intrauterine insemination | Vaginal bleeding | 1 IUP+1CSP | 5 wk and 5 wk 3d | Aspiration of the ectopic gestational sac twice | Live birth at 37 wk by CS  Excision of mass during CS  Selective UAE performed d/t AVM and massive bleeding | 2660 | friable mass 4× 5×2 cm | 5100 |
| Kim 2014  [25] | 34 | 2 | Spontaneous | No complaint | 1IUP+1CSP | 5.5wk | Expectant management | Live birth at 37.3wk by CS with placenta accrete and severe PPH, managed by complete excision of the anterior lower segment and bilateral uterine artery ligation | 2750/2060 | placenta bulging towards the vesicoperitoneal  reflection over the anterior isthmic wall | heavy uterine bleeding |
| Armbrust  2015[26] | 36 | 2 | IVF | No complaint | 1 IUP+1CSP | 7 wk | Laparotomy excision of CSP | Live birth at 37 wk by CS | 2895 | No | 600 |
| Ouyang  2015[4] | 34 | 1 | IVF + ICSI | Vaginal bleeding and abdominal pain | 1IUP+  1CSP | 6wk5d | Expectant management  ( non-viable CSP) | Live birth by ECS at 35+ wk d/t massive hemorrhage caused by complete placenta previa  Excision of mass during CS | 2600 | amorphous mass 2.1×1.4cm, with deciduous tissues l in pathologic examination | massive hemorrhage |
|  | 32 | 1 | IVF | abdominal pain | 1 IUP+1 CSP | 5wk6d | Expectant management  ( non-viable CSP) | TVS guidance D&C d/t IUP termination at 13 w+ | / | / | / |
|  | 38 | 1 | ICSI | abdominal pain | 1 IUP+1 CSP | 7wk4d | Expectant management  ( non-viable CSP) | Second trimester abortion (6 months’ gestation) of IUP | / | / | / |
|  | 28 | 1 | IVF | No complaint | 1IUP+1 CSP | 6wk3d | Expectant management  ( non-viable CSP) | Live birth by CS at 36+ wk d/t PROM | 2900 | Scar mass disappeared at 22 w+ | No data |
|  | 36 | 1 | IVF | Vaginal bleeding | 1 IUP+1CSP | 6wk3d | TVS guidance local KCl | TVS guidance D&C due to IUP  termination at 14 w+ | / | / | / |
|  | 34 | 1 | IVF | Vaginal bleeding and abdominal pain | 1IUP+1 CSP | 7wk1d | Expectant management  ( non-viable CSP) | IUP viable and uneventfully at the time of author report(18 wks’ gestation)  Retained ectopic mass3.2 × 2.7 cm | No data | No data | No data |
| Present case | 33 | 1 | IVF | No complaint | 1 IUP+1CSP | 16wk4d | TAS guidance fetal reduction of CSP with KCl | Live birth by CS at 37 wk6d with placenta previa and placenta accrete, Incomplete uterine rupture  Transfusion of 3.5 packed - red blood cell | 2890 | Dead fetus, complete placenta previa and placenta accreta | 1800 |

CSP: caesarean scar pregnancy; IUP: intrauterine pregnancy; HCSP: heterotopic cesarean scar pregnancy; OHS:ovarian hyperstimulation syndrome; ECS: emergency caesarean section; ICSI: intracytoplasmic sperm injection;D&C:dilatation and curettage; UAE: uterine artery embolization;d/t: due to; AVM: arteriovenous malformation
